# Construction of ubiquitination-related risk model for predicting prognosis in lung adenocarcinoma

**DOI:** 10.1038/s41598-025-92177-4

**Published:** 2025-04-06

**Authors:** Dawei Sun, Xiaohong Duan, Ning Li, Ou Qiao, Yingjie Hou, Zihuan Ma, Siyao Liu, Yanhua Gong, Zichuan Liu

**Affiliations:** 1 School of Disaster and Emergency Medicine, Faculty of Medicine, Tianjin University, No. 92 Weijin Road, Nankai District, Tianjin, 300072 China; 2 Institute of Disaster and Emergency Medicine, Faculty of Medicine, Tianjin University, Tianjin, China; 3 Medical School, Faculty of Medicine, Tianjin University, Tianjin, China; 4 Beijing ChosenMed Clinical Laboratory Co. Ltd, Beijing, 100176 China; 5 School of Pharmaceutical Science and Technology, Faculty of Medicine, Tianjin University, Tianjin, China; 6 Frontiers Science Center for Synthetic Biology (Ministry of Education), Tianjin University, Tianjin, China

**Keywords:** Lung adenocarcinoma, Ubiquitination, Immunotherapy, Prognosis, Biomarker, Chemotherapy, Cancer, Cancer models

## Abstract

Lung adenocarcinoma is the most prevalent lung cancer type. Ubiquitination, a critical post-translational modification process that regulates protein degradation and signaling pathways, has been implicated in various cancers, including LUAD. We aimed to explore the associations between ubiquitination and lung adenocarcinoma. TCGA-LUAD cohort served as the training set. Unsupervised clustering, univariate Cox regression, Random Survival Forests, and least absolute shrinkage and selection operator (LASSO) Cox regression were applied to identify ubiquitination-related genes (URGs), then ubiquitination-related risk scores (URRS) were calculated using gene expression and the univariate Cox’s coefficient. Comparisons between the high and the low URRS group regarding chemotherapy drug response, immune infiltration level, tumor mutation burden (TMB), tumor neoantigen load (TNB), PD1/L1 expression, and enriched pathways were performed. URRS was calculated based on the expression of DTL, UBE2S, CISH, and STC1. Patients with higher URRS had a worse prognosis (Hazard Ratio [HR] = 0.54, 95% Confidence Interval [CI]: 0.39–0.73, *p* < 0.001), and the prognosis of the URRS was further confirmed in 6 external validation cohorts (Hazard Ratio [HR] = 0.58, 95% Confidence Interval [CI]: 0.36–0.93, p_max_ = 0.023). The high URRS group had higher PD1/L1 expression level (*p* < 0.05), TMB (*p* < 0.001), TNB (*p* < 0.001), and TME scores (*p* < 0.001). The IC50 values of various chemotherapy drugs were lower in the high URRS group. In addition, we found that upregulation of STC1, UBE2S, and DTL was associated with worse, while upregulation of CISH was associated with better prognosis. We also performed a reverse transcription-quantitative polymerase chain reaction (RT-qPCR) for validation. In conclusion, the ubiquitination-based signature might serve as a biomarker to help evaluate the prognosis, biological features, and appropriate treatment for patients with lung adenocarcinoma.

**Supplementary Information:**

The online version contains supplementary material available at 10.1038/s41598-025-92177-4.

## Introduction

Non-small cell lung cancers (NSCLCs) are malignant tumors, representing approximately 85% of all cases^1^. Among the various histological subtypes, lung adenocarcinoma (LUAD) is the most frequently diagnosed, accounting for over 40% of NSCLC cases and exhibiting a rising incidence in both smokers and non-smokers^2^. Global epidemiological data indicate that LUAD is a leading cause of cancer-related mortality, with an estimated 1.8 million deaths annually, driven by its high metastatic potential and molecular heterogeneity^3^. Despite advancements in targeted therapies and immune checkpoint inhibitors, the 5-year survival rate for advanced LUAD remains below 20%, underscoring the urgent need for improved prognostic biomarkers^4,5^.

Ubiquitin–proteasome system (UPS), present in all eukaryotes, is a specialized proteolysis system that affects multiple cellular protein processes, including cell signaling, receptor trafficking, cell cycle, and immune response^6^. The ubiquitination process impacts the progression of cancer through multiple pathways, regulating disease progression both in tumor-promoting and tumor-inhibiting pathways. In non-small cell lung cancer (NSCLC), various ubiquitination pathways have been reported to be associated with its occurrence and development^7,8^. For instance, mutations in KRAS are common in NSCLC, and disruptions in the ubiquitination pathways that regulate KRAS protein stability can lead to its prolonged activity. This sustained activity drives oncogenic processes, thereby promoting tumor development and progression^9^. Additionally, the interaction between MetaLnc9 and PGK1 inhibits the ubiquitin-mediated degradation of PGK1, which in turn facilitates the metastasis of lung cancer^10^. Furthermore, the MDM2/MDMX complex, acting as the primary E3 ubiquitin ligase for p53, mediates its degradation. When this pathway is dysregulated in NSCLC, it results in decreased p53 levels, leading to increased cell proliferation and survival^11^. Lastly, the increased stability and activity of HIF-1α are linked to tumor adaptation to hypoxia and metabolic reprogramming. Altered ubiquitination pathways that affect HIF-1α degradation can contribute to these processes, further driving tumor growth and progression^12^. In terms of specific enzymes, various ubiquitination-related enzymes, such as USP22, UBE2S, USP17, and USP10, play crucial roles in regulating signaling pathways like EGFR, Wnt/β-catenin, NF-κB, and AKT, thereby influencing tumor progression, metastasis, and cell cycle control in NSCLC^13–18^. While some enzymes promote cancer malignancy by stabilizing oncogenic proteins or activating pro-tumorigenic signals, others inhibit tumor growth by disrupting these pathways. Moreover, modulating ubiquitin-dependent pathways can facilitate targeted anti-tumor responses by tightly interacting with co-stimulating, co-inhibitory receptors and tumor microenvironment (TME)^19,20^. These studies validate the critical roles of UPS in cancer development, progression, and metastasis.

Many studies have reported that ubiquitination-related proteins can serve as prognostic indicators in LUAD^21,22^. However, as mentioned above, the ubiquitin-proteasome system comprises a large number of ubiquitin molecules, ubiquitin-activating enzyme E1, ubiquitin-binding enzyme E2, ubiquitin-ligase E3, and deubiquitinase. In the ubiquitin-proteasome pathway, proteasome is also required. Thus, the influence of the ubiquitin-proteasome system on tumor progression should be the result of the cooperation of these factors. There remains a knowledge gap regarding which ubiquitination-related factors contribute most significantly to disease prognosis. Therefore, bioinformatics methods could be applied to assess the relevance of ubiquitination-related genes (URGs) with TME and their prognostic values in lung adenocarcinomas.

This study identified ubiquitination-related molecular subtypes based on the unsupervised clustering algorithm using the TCGA lung adenocarcinoma (TCGA-LUAD) cohort. A comparison was made between two subtypes of ubiquitination in terms of gene mutation frequency and tumor mutation burden (TMB). Differential genes were screened out between two ubiquitination subtypes. Based on four prognostic genes, identified using least absolute shrinkage and selection operator (LASSO) Cox regression, Random Survival Forest, and Univariate Cox Regression algorithms, a ubiquitination-related risk score (URRS) prognostic model was further developed. Finally, the association between URRS and prognostic performance, immune status, and treatment response was evaluated.

## Methods

**Fig. 1:**
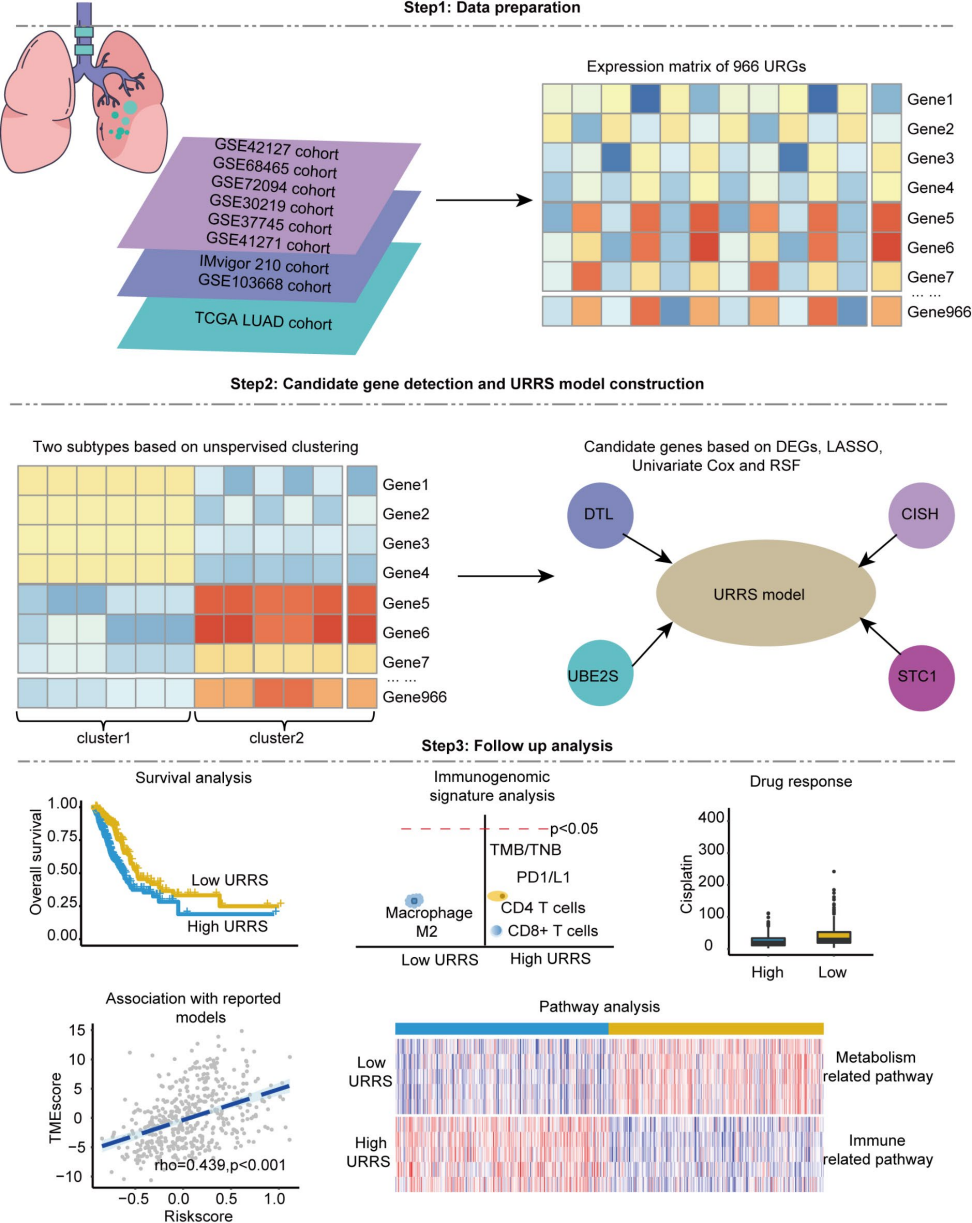
Workflow chart.

The workflow of this study is shown in Fig. 1.

### Data source

Nine hundred and sixty-six ubiquitination-related genes (URGs), including ubiquitin-activating enzymes (E1s), ubiquitin-conjugating enzymes (E2s), and ubiquitin-protein ligases (E3s), were collected from iUUCD 2.0 database (http://iuucd.biocuckoo.org/)^23^.

The gene expression profiles and the corresponding clinical datasets of lung adenocarcinoma were collected from the Gene Expression Omnibus (GEO) and the Cancer Genome Atlas (TCGA). TCGA-LUAD datasets were obtained to reveal the feature genes. Somatic mutations, including somatic SNV/indel and copy number variation (CNV), were respectively downloaded from the cBioPortal database (https://www.cbioportal.org/study/summary?id=luad_tcga_pan_can_atlas_2018) and UCSC Xena (https://xenabrowser.net/). For processing the data, we first retained only the cancerous tissues in the dataset, excluding formalin-fixed samples and recurrent tissues. Subsequently, patients with a survival time of fewer than 3 months were filtered out. Among the remaining samples, we further selected those with available RNA-seq data for our analysis. Seven GEO datasets, including one dataset (GSE103669)^24^ treated with immune therapy and six datasets (GSE30219^25^, GSE37745^26^, GSE41271^27^, GSE42127^28^, GSE68465^29^ and GSE72094^30^) were applied to validate the performance. In addition, the gene expression data and detailed information of patients treated with anti-PD-L1 agents named IMvigor210 cohort were downloaded using the R package “IMvigor210CoreBiologies”^31^. Patients in all datasets whose survival time was lower than 3 months were excluded for further analysis. Supplementary Table 1 provides comprehensive information regarding all datasets, while Supplementary Table 2 presents the proportion of censored data across all datasets.

### Consensus clustering analysis of URGs

The unsupervised clustering “KM” method combined with Euclidean distance was applied to identify distinct molecular subtypes based on URG expression. The “ConsensusClusterPlus” R package^32^ using the “ConsensusClusterPlus” function (maxK = 5, reps = 1000, pItem = 0.8, pFeature = 1, clusterAlg="km”, distance="euclidean”) determined the number of clusters in the TCGA-LUAD cohort and repeated 1000 times to ensure classification stability. We explored the prognostic performance and other clinical features, including age, survival status, sex, and stage of molecular subtypes. Differently expressed URGs (adjusted p-value < = 0.05 and |log2FC| >=0.8) in different molecular subtypes were identified using the “limma” R package^33^.

### Somatic mutation analysis

Silent mutations were excluded for subsequent analysis, and the “Maftools” R package^34^ was applied to analyze and visualize the mutation landscape. In the GDC GISTIC copy number dataset, CNVs that equaled 2 and − 2 were considered amplification and deep deletion. The difference in mutation frequency in different molecular subtypes was also compared.

### Calculation of the ubiquitination-related risk score

Prognostic ubiquitination-related genes were detected and overlapped by Univariate Cox regression analysis, Random Survival Forest algorithm (variable importance > 0.25)^35^ using “randomForestSRC” package with “rfsrc” function (ntree = 100, nsplit = 5, importance = TRUE, tree.err = TRUE) and LASSO Cox regression algorithm^36^ with “cv.glmnet” function (family=’cox’, type.measure = ‘deviance’) based on differently expressed URGs. We chose Random Survival Forest for its robustness in handling high-dimensional data and complex interactions, while LASSO was used for feature selection and regularization to enhance model interpretability and prevent overfitting. Ubiquitination-related risk scores (URRS) were calculated based on Multivariate Cox regression analysis, and the formula was as follows:

$$\:Risk\:score=\:\sum\:{\beta\:}_{RNA}*{Exp}_{RNA}\:\:\:\:\:\:\:\:\:\:\:\:\:\:\:\:\:\:\:\:\:\:\:\:\:\:\:\:\:\:\:\:\:\:\:\:\:\:\:\:\:\left(1\right)$$

In this formula, β_RNA_ represents the coefficient in the Multivariate Cox regression analysis of the differently expressed URGs, while Exp_RNA_ represents the expression of the differently expressed URGs.

According to the median value, all the patients were divided into high and low URRS groups. Time-dependent ROC curves were further used to evaluate the efficiency and accuracy of URRS in predicting outcomes at one-, three- and five-year endpoint events.

### Immune infiltration analysis

Immune infiltration in the tumor microenvironment of lung adenocarcinoma was carried out by the CIBERSORT algorithm^37^. Stromal score, immune score, and ESTIMATE score were evaluated via the ESTIMATE (Estimation of Stromal and Immune cells in Malignant Tumor tissues using Expression data) algorithm^38^. Tumor mutation burden (TMB) score, TIDE score, and TME score were also applied to predict the immunotherapeutic response to URRS in lung adenocarcinoma. TME score was used to predict the ICB efficacy and was calculated by the “TMEscore” R package^39^. TIDE score measuring the ICB efficacy was obtained from the TIDE website (http://tide.dfci.harvard.edu/). The tumor neoantigen load and immune subtypes were extracted from the research. In addition, the APOBEC enrichment score based on mutation landscape was calculated by the “Maftools” R package^34^. Tumor neoantigen burden (TNB) and immune subtypes were retrieved from Thorsson’s study^40^.

### Drug response prediction and enrichment pathway analysis

All gene sets from the Kyoto Encyclopedia of Genes and Genomes (KEGG)^41^ were downloaded from the MSigDB database^42^. Gene Set Variation Analysis (GSVA) was carried out based on the single-sample gene set enrichment analysis (ssGSEA) method^43^. The half maximal inhibitory concentration (IC50) value was estimated by the “oncoPredict” R package^44^ based on the expression profile and pharmacogenomic data from Genomics of Drug Sensitivity in Cancer (GDSC) database^45^. The GDSC database includes data on 198 different drugs. We focused on drugs that are commonly used in the treatment of LUAD which is indicated in the CIViC database^46^.

### Expression profile and prognosis analysis in pan-cancer datasets

Pan-cancer gene expression and prognosis analysis of ubiquitination-related prognostic genes were implemented using Gene Set Cancer Analysis (GSCA), a Multi-omics online analysis tool based on the TCGA datasets (http://bioinfo.life.hust.edu.cn/web/GSCALite/). The difference in expression profile between the normal and the tumor was estimated by the GEPIA2 website (http://gepia.cancer-pku.cn/*).*

### Cell culture and reverse transcription-quantitative polymerase chain reaction (RT-qPCR)

Three human lung cancer cell lines, A549, HCC-827, and NCI-H1299, and one human lung bronchial epithelial cell line, BEAS-2B, were obtained from the American Type Culture Collection (ATCC). HCC-827 and NCI-H1299 cells were cultured in RPMI 1640 medium (Gibco); A549 cells were cultured in F12K medium (Hyclone); BEAS-2B cells were cultured in DMEM medium (Gibco). Hyclone); BEAS-2B cells were cultured in DMEM medium (Gibco). All the cultures medium were supplemented with 5% fetal bovine serum (FBS, Gibco). In addition, trypsin-EDTA (Gibco) was used to disperse the cells during cell passage and collection. Cell cultures were maintained at a constant temperature of 37 °C and 5% CO2 to provide optimal conditions for cell growth and viability.

RT-qPCR experiments were performed to verify the differential expression of four URGs (including DTL, UBE2S, CISH, and STC1) between lung cancer tumor cell lines (A549, HCC-827, and NCI-H1299) and a normal lung bronchial epithelial cell line (BEAS-2B). Total RNA was extracted using Triquick Reagent (Sangon Biotech) according to the manufacturer’s protocol, and RNA was reverse transcribed to cDNA in 20 µl of reaction mixture using HiScript III RT kit (TaKaRa, Shiga, Japan). The resulted cDNA was then subjected to qPCR using a qPCR kit (ChamQ Universal SYBR qPCR Master Mix) (Yeasen Biotechnology) in 20 µl of reaction mixture, with three replicates per gene per sample. The expression of the target genes was detected using an ABI7300 Fast instrument (Thermo Fisher Scientific, USA). The expression level of each gene was normalized to the reference gene GAPDH and analyzed using the 2^(-ΔΔCT) method, which is widely accepted in scientific research (ΔCT = CT (target gene) - CT (reference gene), ΔΔCT = ΔCT (NSCLC cells) - ΔCT (normal lung cells)). Primer sequences are shown in Table 1 and CT values for RT-qPCR are shown in Supplementary Table 3.

**Table 1:** Primer sequences utilized for RT-qPCR experiment in this study.

Gene	Forward Primer	Reverse Primer	Size
GAPDH	ACAACTTTGGTATCGTGGAAGG	GCCATCACGCCACAGTTTC	101 bp
DTL	TCACTGGAATGCCGTCTTTGA	CTCACCAGCTTTTACGTCCC	106 bp
UBE2S	ACAAGGAGGTGACGACACTGA	CCACGTTCGGGTGGAAGAT	216 bp
CISH	GAACTGCCCAAGCCAGTCAT	GCTATGCACAGCAGATCCTCC	131 bp
STC1	CACGAGCTGACTTCAACAGGA	GGATGTGCGTTTGATGTGGG	110 bp

### Statistical analysis

The statistical analyses used the following R packages: ‘randomForestSRC’ for Random Survival Forest analysis, ‘glmnet’ for LASSO regression, and ‘survival’ for Cox proportional hazards models. All the survival curves were measured via the Kaplan-Meier method between different groups, and the log-rank test compared the difference in survival time. The Wilcoxon test estimated the P-value between the two groups, and a p-value < = 0.05 was considered statistically significant. All the statistical analysis was conducted using the R software (version 4.1.2). The variables (genes) included in the URRS model were selected based on their importance in the Random Survival Forest analysis (variable importance > 0.25) and their significant association with overall survival in the univariate Cox regression analysis (*p* < 0.05). These criteria ensured that only the most prognostically relevant genes were included in the model. To account for multiple comparisons, the false discovery rate (FDR) correction was applied using the Benjamini-Hochberg method. This ensured that the reported p-values were adjusted for the number of tests performed, reducing the likelihood of type I errors.

## Results

### Identification of ubiquitination subtypes in LUAD

**Fig. 2:**
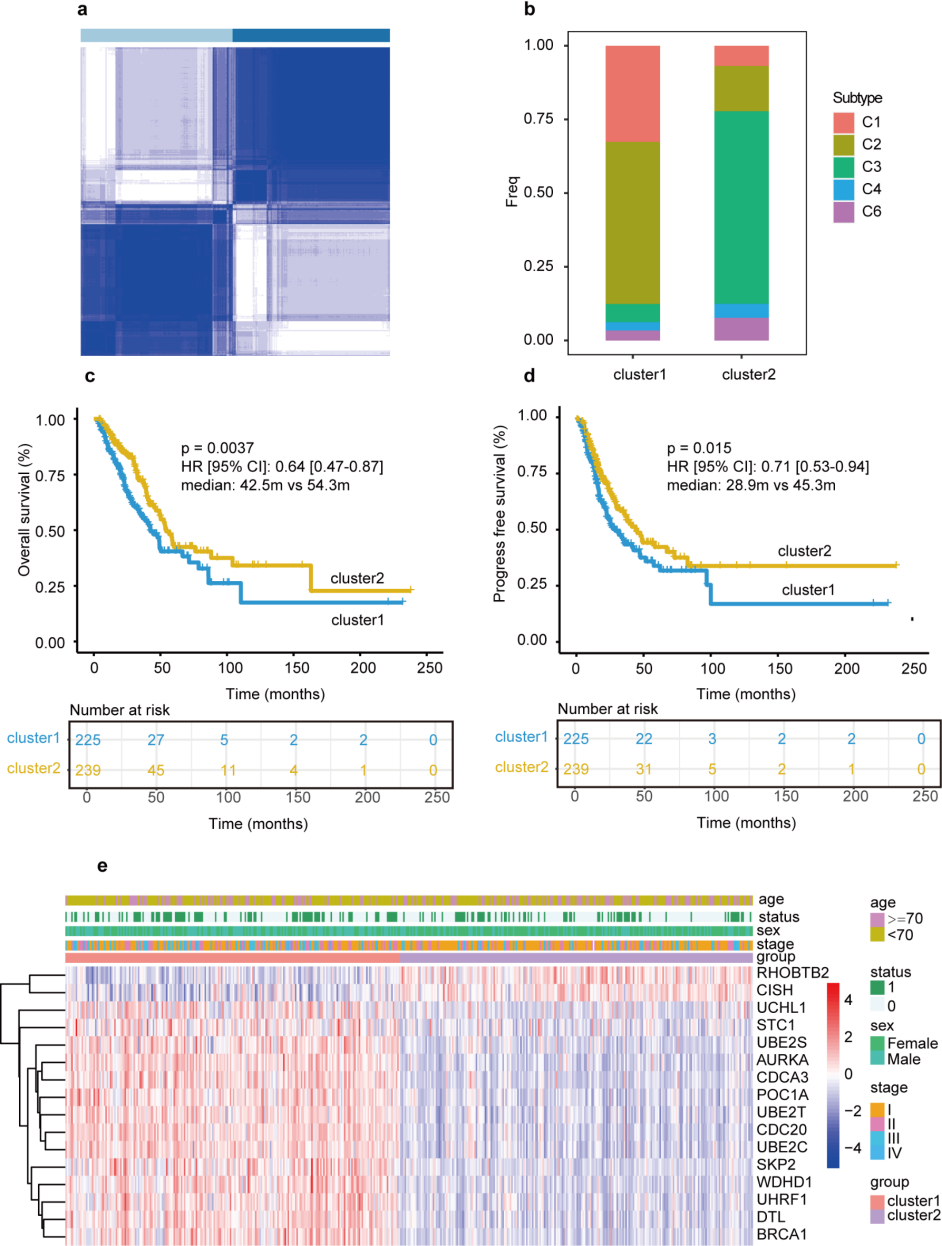
Characteristics of ubiquitination-related clusters in TCGA-LUAD cohort. **(a)** The cluster diagram of subtype based on the 996 ubiquitination-related gens (URGs). **(b)** Percentage of subtype obtained from previous report between two clusters. **(c)** Overall survival (OS) Kaplan-Meier (KM) curve in two clusters. **(d)** Progress free survival (PFS) KM curve between two clusters. **(e)** The heatmap of 16 differential URGs and clinical features between two clusters.

Applying the unsupervised clustering approach, two distinct molecular subtypes were identified according to the expression levels of URGs from the TCGA (Fig. 2a). The results above were further supported by cluster2 having a larger percentage of the C3 subtype, which corresponded with improved clinical outcomes (Fig. 2b). Patients with lung adenocarcinoma in cluster2 had significantly longer overall survival (Fig. 2c, *p* = 0.004, median months: 42.5 m vs. 54.3 m) and progression-free survival (Fig. 2d, *p* = 0.015, median months: 28.9 m vs. 45.3 m). Compared with cluster1, expressions of CISH, RHOBTB2 were higher in cluster2, while UCHL1, STC1, UBE2S, AURKA, CDCA3, POC1A, UBE2T, CDC20, UBE2C, SKP2, WDHD1, UHRF1, DTL and BRCA1 were lower in cluster2 (Fig. 2e). Furthermore, patients of cluster2 had a statistically higher proportion of female and alive outcomes (Fig. 2e).

### Somatic mutation landscape of URGs

**Fig. 3:**
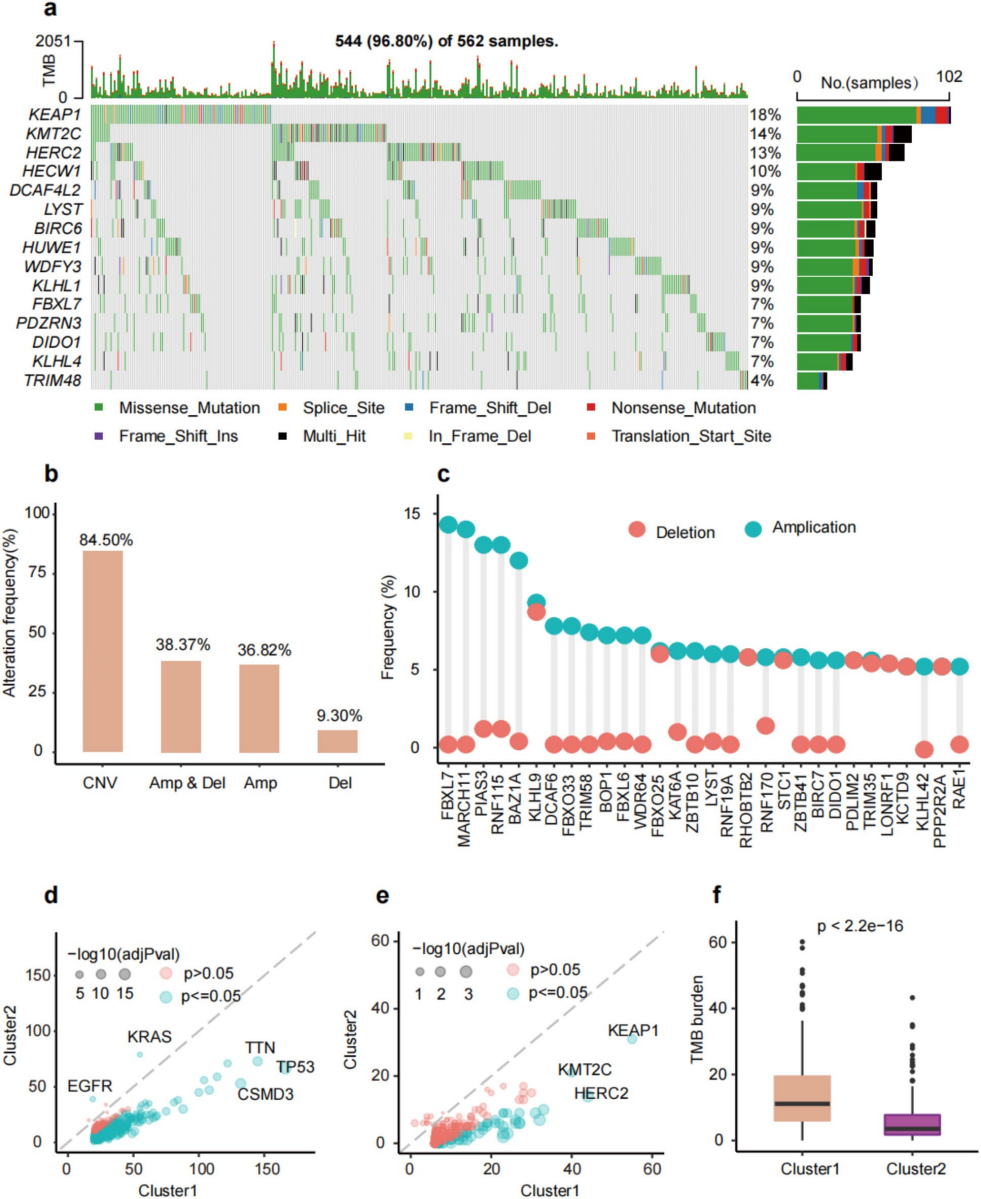
Mutation profiles of URGs in TCGA-LUAD cohort. **(a)** Oncoplot of ubiquitination-related gene non-silent somatic mutations. **(b)** Percentage of copy number variations (CNVs). **(c)** The frequency of CNVs in top 30 URGs. **(d)** The mutation frequency of all genes between two clusters. **(e)** The mutation frequency of URGs between two clusters. **(f)** The difference of tumor mutation burden (TMB) between two clusters.

To study the genomic features of URGs in LUAD, we visualized the SCNA and mutation frequency of LUAD patients in the TCGA cohort. About 96.80% of patients’ ubiquitination-related genes mutated, and the top 15 gene mutation frequency ranged from 4 − 18%. KEAP1, KMT2C, HERC2, and HECW1 genes showed a higher frequency (above 10%, Fig. 3a). In addition, about 84.50% of LUAD patients had at least one SCNA of URGs, 38.37% of patients with both gene amplification and deep deletion, 36.82% of patients with only amplification and 9.30% of patients with only deep deletion (Fig. 3b). FBLX7, MARCH11, PIAS3, RHF115, and BAZ1A showed higher amplification alteration frequency (above 10%, Fig. 3c). Interestingly, we found lower mutation frequency in most genes except for EGFR and KRAS genes (Fig. 3d) and lower ubiquitination-related gene mutation frequency in cluster2 (Fig. 3e) and lower TMB level (Fig. 3f). In conclusion, these findings revealed that dysregulation of URGs might be caused by mutation and lead to poor prognosis.

### Construction and validation of ubiquitination-related signature for the prognostic prediction in LUAD

**Fig. 4:**
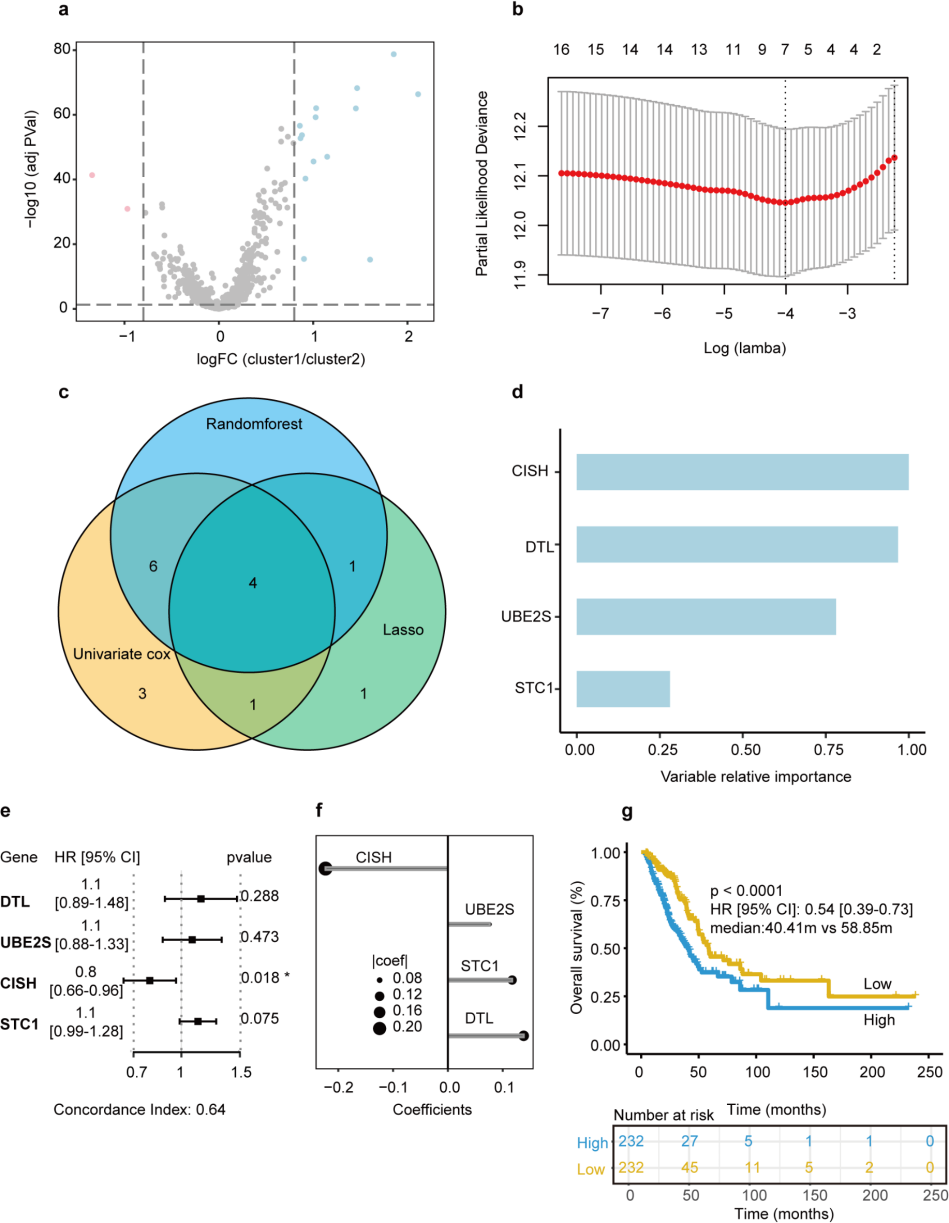
Construction of ubiquitination-related risk scores (URRS) model. **(a)** Volcano plot between two clusters. **(b)** least absolute shrinkage and selection operator (LASSO) coefficient profiles 16 selected URGs in the 10-fold cross-validation. **(c)** The overlap genes based on Random Survival Forest, Univariate Cox regression and LASSO Cox regression algorithms. **(d)** The variable relative importance of overlapped URGs. **(e)** The forest plot of the associations between OS and 4 prognostic URGs. **(f)** The coefficient of 4 URGs in the Multivariate Cox regression model. **(g)** The KM curve of OS between two URRS groups.

16 differentially expressed genes (DEGs) were selected (|log2FC|>=0.8, adjusted p-value < = 0.05) in the two subclusters (Fig. 4a). Through Univariate Cox Regression, Random Survival Forest (relative importance > 0.25) and LASSO COX Regression analysis, 4 ubiquitination-related genes (DTL, STC1, CISH, UBE2S) were selected (Fig. 4b and d) to create the URRSmodel. The forest plot also illustrated the associations between the expression levels of 4 ubiquitination-related signatures and the OS of patients (Fig. 4e). High expression levels of DTL, STC1, and UBE2S contributed to a poorer prognosis, while high expression levels of CISH played the opposite roles. URRS was established using the formula: URRS= (0.1386*DTL) + (0.07634*UBE2S) + (0.11723*STC1) + (-0.22321*CISH) (Fig. 4f). Based on the median URRS, we split up all the patients into the high URRS group and the low URRS group and found that the low URRS group had a longer overall survival than the high URRS group (p < = 0.001, median months:40.41 m vs. 58.85 m, Fig. 4g).

**Fig. 5:**
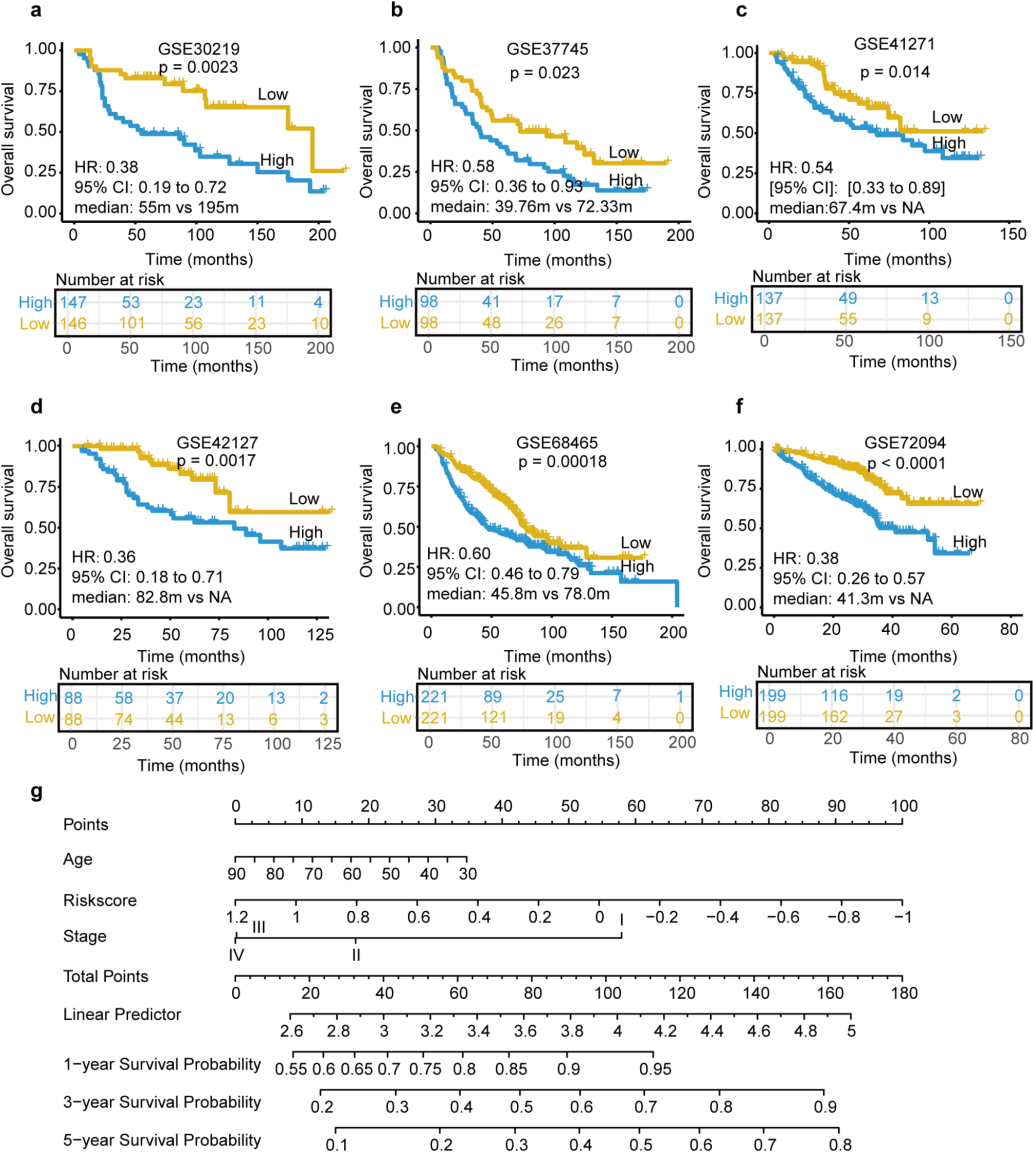
Validation of URRS in six GEO cohorts and the Nomogram of LUAD patients in TCGA-LUAD. **(a)** GSE30219 cohort. **(b)** GSE37745 cohort. **(c)** GSE41271 cohort. **(d)** GSE42127 cohort. **(e)** GSE68465 cohort. **(f)** GSE72094 cohort. **(g)** Nomogram of patients in TCGA-LUAD.

The survival ROC curves predicted by the ubiquitination signature showed that the AUCs in the TCGA-LUAD cohort at the 1-year (AUC = 0.700), 3-year (AUC = 0.651) and 5-year (AUC = 0.587) time points (Figure S1a). Ubiquitination-related signature was also validated in other six cohorts: GSE30219 (*p* = 0.0023, Fig. 5a, Figure S1b), GSE37745 (*p* = 0.023, Fig. 5b, Figure S1c), GSE41271 (*p* = 0.014, Fig. 5c, Figure S1d), GSE42127 (*p* = 0.0017, Fig. 5d, Figure S1e), GSE68465 (*p* = 0.00018, Fig. 5e, Figure S1f) and GSE72094 cohorts (*p* < 0.0001, Fig. 5f, Figure S1g). A Nomogram (Fig. 5g) and a forest plot (Figure S1h) were drawn to better visualize the predicted performance of URRS on the prognosis of LUAD patients in TCGA-LUAD. Figures S1i to k show the net clinical benefit for patients with os of one, three, and five years, respectively. Figure Sl demonstrates the calibration ability of the nomogram, with a c-index of 0.71.

### Ubiquitination signature-related genes in multiple cancers

**Fig. 6:**
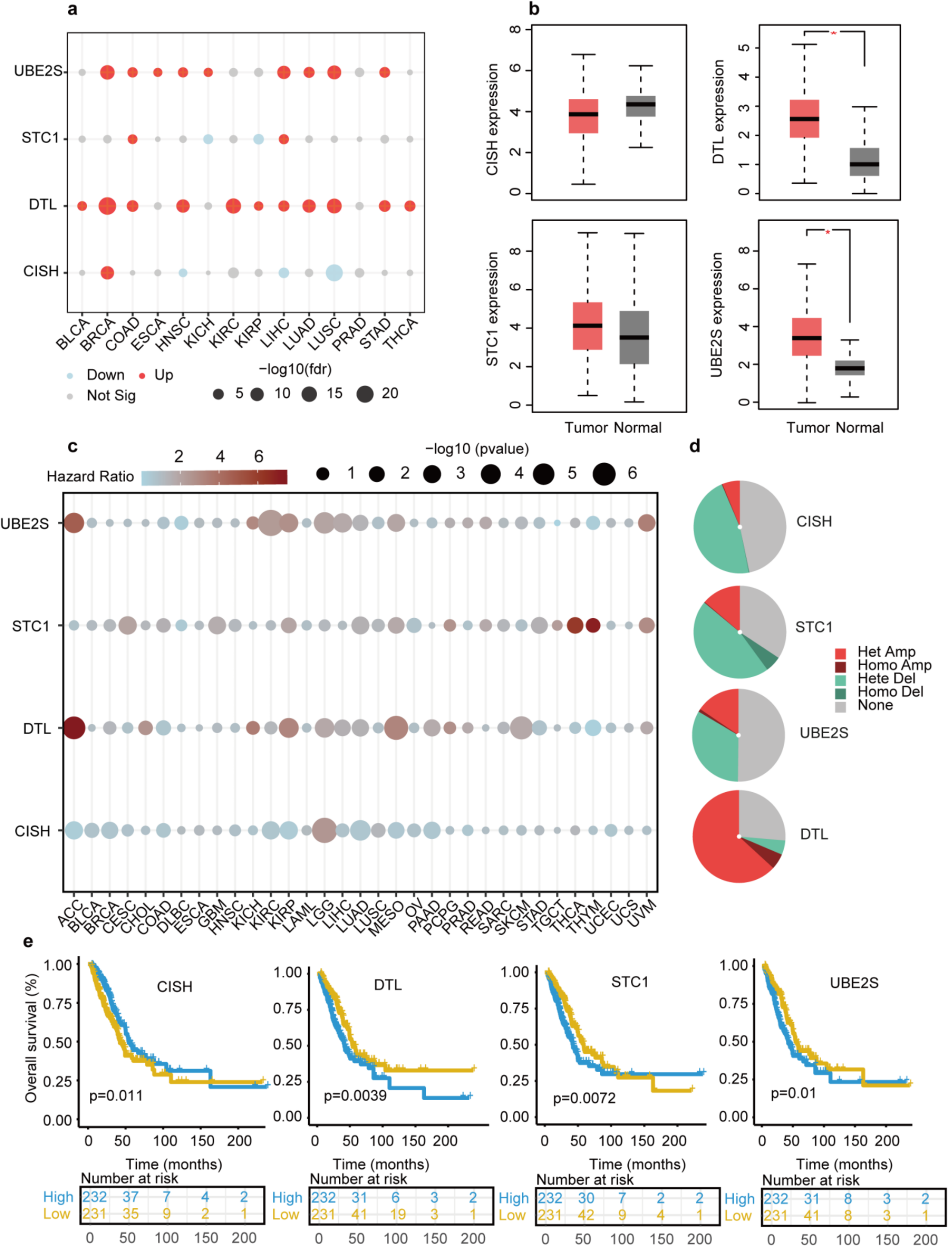
Expression, survival and CNV frequency profiles of 4 URGs in multiple cancers. **(a)** Expression profiles of 4 URGs between normal and tumor samples in multiple cancers. **(b)** Expression profiles of 4 URGs between normal and tumor samples in TCGA-LUAD cohorts using GEPIA2. **(c)** The survival profiles of 4 URGs between tumor and normal samples in multiple cancers. **(d)** CNV profiles of 4 URGs in TCGA-LUAD cohort. **(e)** OS KM curves of 4 URGs in TCGA-LUAD cohort.

The GSCA online tool was utilized to analyze the expression patterns of four genes related to the ubiquitination signature across multiple cancer types, requiring a minimum of three matched tumor and normal samples. (Fig. 6a). For most cancers, the expression levels of UBE2S and DTL were increased in tumor samples, while STC1 expression was only increased in tumor samples of COAD and LIHC, but decreased in KICH and KIRP. CISH was down-regulated in tumor samples of LUSC, LIHC, and HNSC while up-regulated in BRCA. GEPIA2 analysis in LUAD patients indicated that DTL and UBE2S were significantly up-regulated in tumor samples. CISH and STC1 were not statistically different, according to results from GSCA online tools (Fig. 6b). In addition, we found that CISH (*p* = 0.0399, Figure S2a) had a higher expression level in early stages. DTL (*p* < 0.001, Figure S2b), STC1 (*p* < 0.001, Figure S2c), and UBE2S (*p* = 0.0478, Figure S2d) showed higher expression level in advanced stages. Pan-cancer survival analysis indicated that the DTL, STC1, and UBE2S played damaged roles in multiple cancers, while CISH showed the opposite role (Fig. 6c). DTL amplification accounts for most of all types, while most STC1 and CISH CNAs were deletions (Fig. 6d). The Kaplan-Meier plot from GEPIA2 validated that up-regulated expression levels of DTL, STC1, and UBE2S had worse overall survival, while CISH had a better overall survival (Fig. 6e). Methylation correlation analysis showed that the methylation level of UBE2S (rho=-0.37, FDR < 0.001) and CISH (rho=-0.52, FDR < 0.001) were negatively correlated with its gene expression level, however, STC1 (rho=-0.08, *p* = 0.0596) and DTL (rho = 0.07, *p* = 0.133) were not statistically correlated (Figure S2e).

### Risk score-based treatment strategy in LUAD

**Fig. 7:**
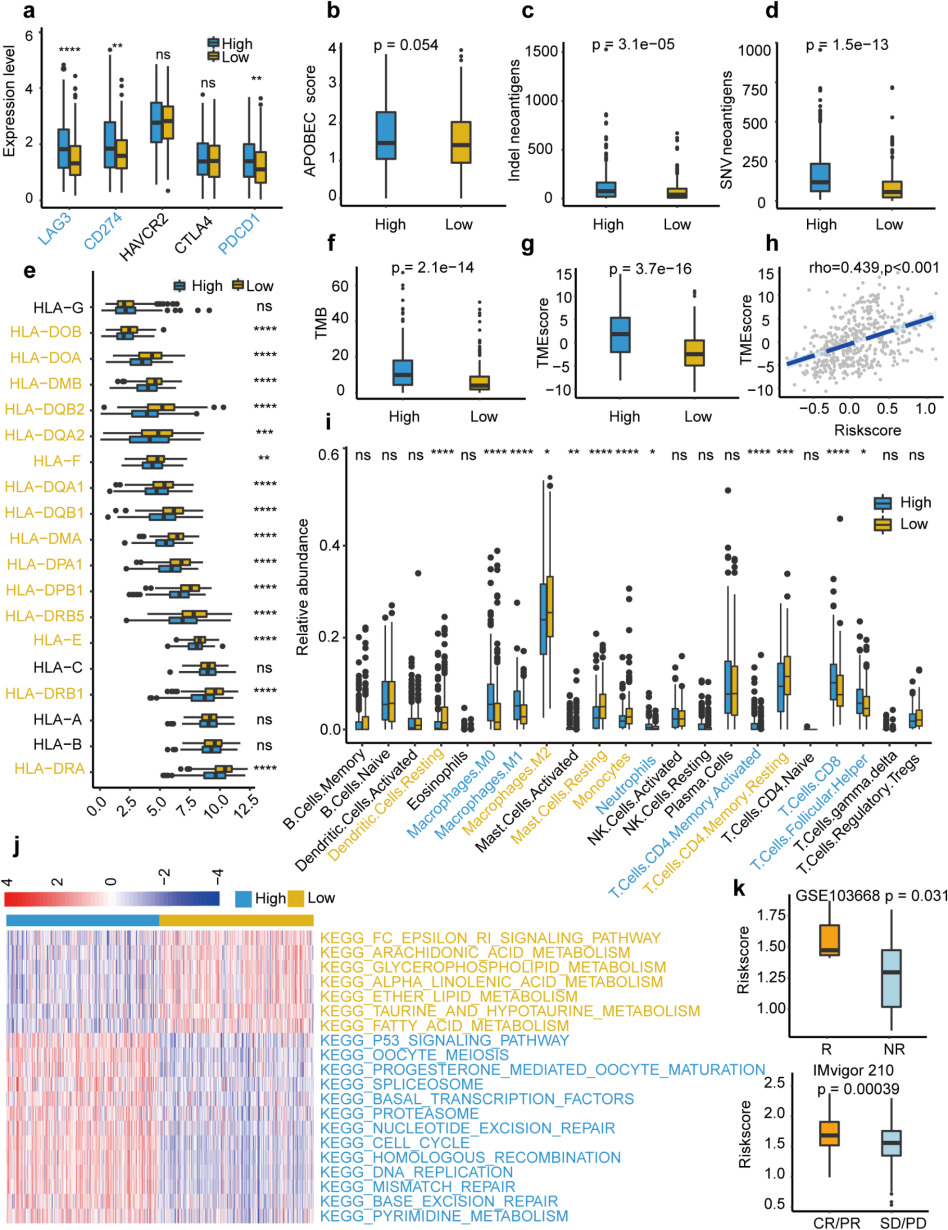
Immune-related profiles in TCGA-LUAD cohort. **(a)** The expression of immune checkpoint genes between two URRS groups. **(b)** The APOBEC mutation score between two URRS groups. **(c-d)** The tumor neoantigen load (TNB) between two URRS groups. **(e)** The expression of HLA-related genes between two URRS groups. **(f)** Tumor mutation burden (TMB) between two URRS groups. **(g)** Tumor microenvironment (TME) score between two URRS groups. **(h)** Correlation between TME score and URRS. **(i)** Immune infiltration between two URRS groups. **(j)** Top 20 enrichment pathways between two URRS groups. **(k)** Immunotherapy response between two URRS groups in GSE103669 and IMvigor 210 cohorts.

The expression of five immune checkpoint genes (LAG3, CD274, HAVCR2, CTLA4, and PDCD1) was compared between high and low URRS groups. The results indicated that LAG3, CD274, and PDCD1 were significantly up-regulated in the high URRS group (Fig. 7a). APOBEC mutational signature (*p* = 0.054, Fig. 7b), tumor neoantigen burden (TNB, *p* < 0.001, Fig. 7c and d), and tumor mutation burden (TMB, *p* < 0.001, Fig. 7f) were also enriched in the high URRS group. Furthermore, the low URRS group had a higher HLA-related gene expression than the high URRS group (Fig. 7e). In addition, we found that TME scores were statistically correlated with URRS, and the high URRS group had higher TME scores (*p* < 0.001, rho = 0.439, Fig. 7g and h). TIDE score analysis indicated that the high URRS group had a lower dysfunction score and higher exclusion score (Figure S3a-S3b). ESTIMATE algorithm was also applied based on the TCGA cohort and indicated that there were significantly higher immune (Figure S3**c**) and estimate scores (Figure S3d) in the low URRS group and no difference in the stromal score (Figure S3e). Immune infiltration was also conducted and results suggested that M0/M1 Macrophages, Neutrophils, activated CD4 T cells, CD8 T cells, and Follicular helper T cells were abundant in the high URRS group while resting Dendritic cells, M2 Macrophages, resting Mast cells, Monocytes and resting memory CD4 T cells showed the opposite trends (Fig. 7i). Immune-related pathways, including homologous recombination, mismatch repair, and nucleotide excision repair pathways were enriched in the high URRS groups, while metabolism-related pathways were enriched in the low URRS group (Fig. 7j). Finally, due to relatively few immunotherapy cohorts in LUAD, GSE103669 (*p* = 0.031) and IMvigor 210 (*p* < 0.001) cohorts were applied to validate the immunotherapy response, and high URRS group had a higher proportion of immune response patients (Fig. 7k).

**Fig. 8:**
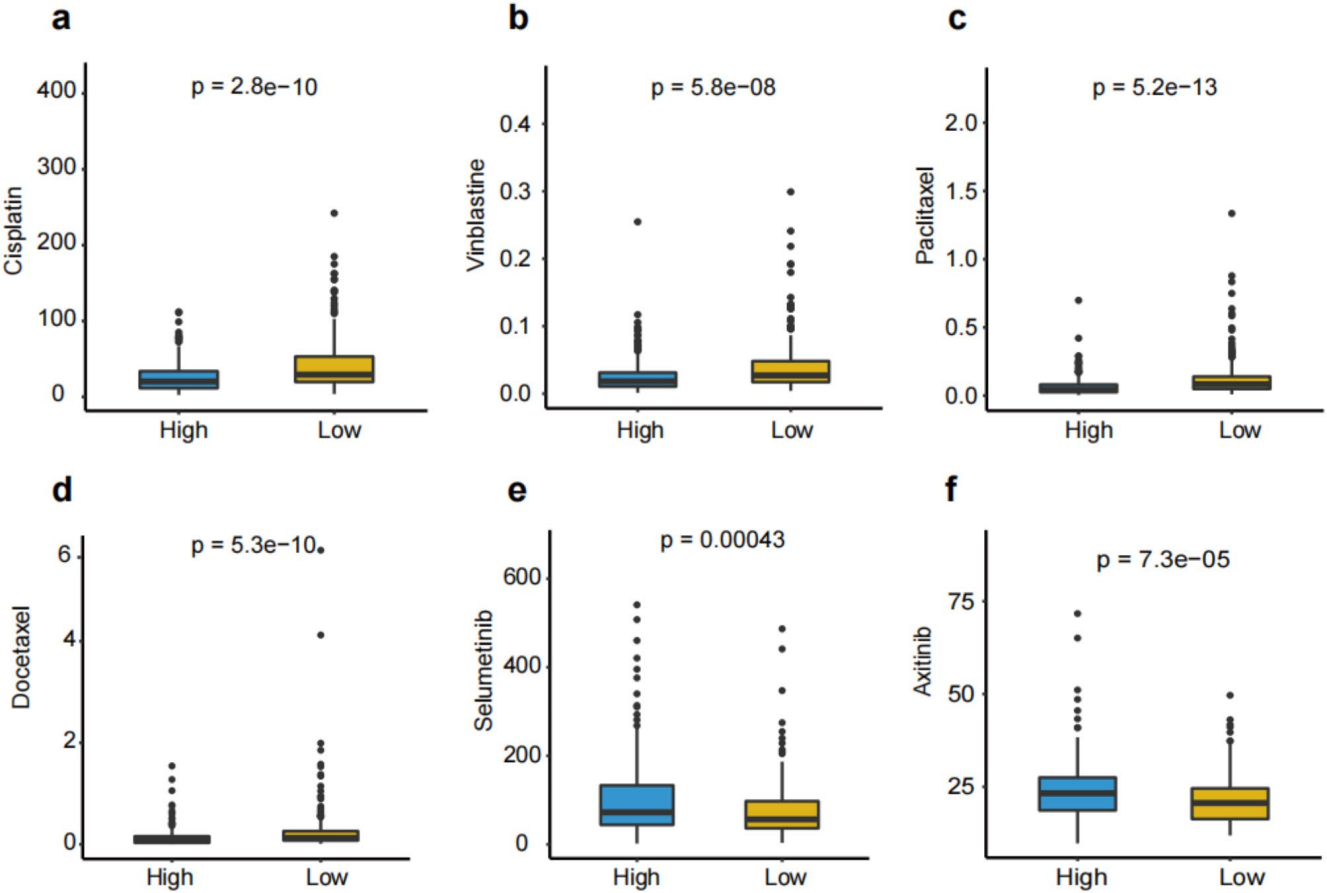
IC50 values of six chemotherapy or target drugs. **(a)** IC50 values of Sorafenib, **(b)** Axitinib, **(c)** Afatinib, **(d)** Pictilisib, **(e)** Selumetinib, **(f)** and Dabrafenib.

In addition, based on the recommendations from the CIViC database, IC50 values of common chemotherapeutic drugs for LUAD have evaluated the drug sensitivity in two groups. We found that the IC50 values of Cisplatin (*p* < 0.001, Fig. 8a), Vinblastine (*p* < 0.001, Fig. 8b), Paclitaxel (*p* < 0.001, Fig. 8c), Docetaxel (*p* < 0.001, Fig. 8d) were lower in the high URRS group, while Selumetinib (*p* < 0.001, Fig. 8e) and Axitinib (*p* < 0.001, Fig. 8f) were lower in the low URRS group. The above results implied that the high URRS group was sensitive to chemotherapy drugs.

### The mRNA and protein expression difference of four URGs between tumor and normal cells in lung cancer

**Fig. 9:**
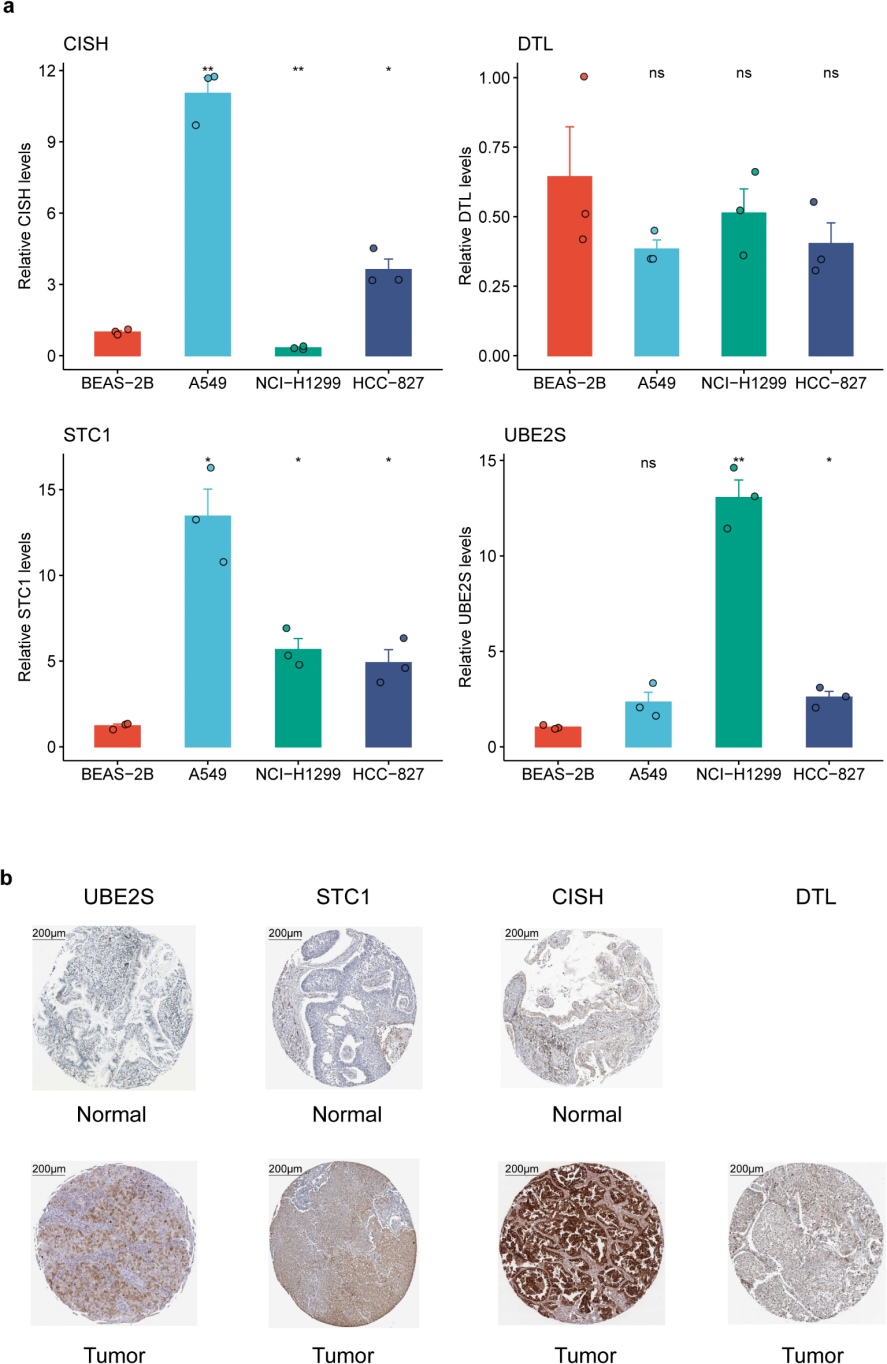
Comparison analysis of the expression of three URGs between tumor and normal samples at RNA and protein levels. **(a)** RNA expression differences of seven URGs between two tumor cell lines and one normal cell line. **(b)** IHC image acquired from HPA database showing the protein expression differences between tumor and normal tissues. IHC: Immunohistochemistry. P value: ns, not significant; * < 0.05; ** < 0.01.

RNA expression validation conducted in three human lung cancer cell lines, A549, HCC-827, and NCI-H1299, along with a human lung bronchial epithelial cell line (BEAS-2B), revealed significant expression differences between normal and tumor cells (Fig. 9a). It was observed that the expression of the STC1 gene was significantly higher in all three lung cancer cell lines compared to the BEAS-2B cell line, aligning with data from the TCGA dataset (Fig. 6b). Similarly, the UBE2S gene also exhibited higher expression in lung cancer cell lines. In contrast, the CISH gene showed notably lower expression in the H1299 cell line compared to the BEAS-2B cell line. Concurrently, the DTL gene demonstrated a trend of lower expression in lung cancer cell lines, consistent with the findings presented in Fig. 6b. Protein expression results obtained from the Human Protein Atlas (HPA) database were consistent with mRNA findings, with the exception that images for DTL in normal tissues were missing in the HPA database. This consistency underscores the significant differences between NSCLC tumors and normal tissues (Fig. 9b).

## Discussion

Although there are several treatment options for lung cancer, a significant number of patients still demonstrate drug resistance^47^. Insufficient treatment options could sometimes lead to overtreatment or undertreatment. In addition, ubiquitination has been reported to serve as a degradation mechanism of proteins involved in cell processes such as activation of NFκB inflammatory response and DNA damage repair in lung cancers^48^. For the above reasons, establishing reliable prognostic biomarkers associated with ubiquitination-related genes to stratify LUAD patients is urgently needed. The URRS model we developed has potential clinical implications for guiding personalized treatment strategies in lung adenocarcinoma. For instance, patients in the high URRS group, who are associated with worse prognosis, higher tumor mutation burden (TMB), and elevated immune-related markers, may benefit more from immunotherapy. Moreover, patients in the high URRS group might be more responsive to conventional chemotherapy. The URRS model thus provides a framework for selecting optimal treatment strategies based on individual risk scores.

The study applied several bioinformatics algorithms to identify a robust and stable ubiquitination-related signature. Firstly, patients with lung adenocarcinoma were clustered into two clusters based on expressions of URGs and mutation status was evaluated. Most URGs, including 96.80% of SNV/INDEL and 84.50% CNV, occurred in lung adenocarcinoma patients. Furthermore, compared to cluster2, cluster1 had a shorter overall survival and a higher frequency of gene alterations, including ubiquitination-related and TP53 gene mutations. Therefore, ubiquitination-related gene mutations might be closely associated with ubiquitination-related gene expressions. A worse prognosis for survival was found to correlate with KEAP1, one of the ubiquitination-related gene co-mutations and TP53 gene mutations in lung cancers^49–54^.

Four URGs, including DTL, UBE2S, STC1, and CISH, were detected to construct the URRS model. Patients were separated by URRS, the low URRS group had a more prolonged overall survival which was also validated in six lung adenocarcinoma cohorts. We discovered that lung cancer patients with higher DTL, UBE2S, and STC1 gene expression had worse prognoses, while the CISH gene showed the opposite trend, which was validated in the previous reports^14,15,55,56^. In addition, RT-qPCR results also indicated that the expression of these four URGs differed between lung cancer cell lines and normal lung cell lines. The expression of the CISH gene in the H1299 lung cancer cell line was significantly lower than that in the normal cell line; the expression of the STC1 and UBE2S genes were both significantly elevated in all three lung cancer cell lines; whereas, the DTL gene exhibited a lower trend than normal cell lines (Fig. 9a), and these expression differences were similarly found in the TCGA dataset (Fig. 6b). At the same time, DTL had a higher proportion of gene amplification, which suggested that expression change of UBE2S and CISH might be associated with DNA methylation and upregulated expression of DTL might be associated with copy number variations.

The four genes that constructed the URRS model in this study have been reported for their important roles in disease progression and therapeutic resistance of LUAD. DTL, an E3 ubiquitin ligase, regulates various cellular processes by promoting the ubiquitination and subsequent degradation of specific substrates. In LUAD, high expression of DTL is associated with enhanced tumor proliferation and invasiveness. DTL may facilitate cancer progression by regulating the ubiquitin-dependent degradation of p53, a crucial tumor suppressor protein^56^. UBE2S activates the NF-κB signaling pathway by binding to IκBα, thereby promoting the metastasis of lung adenocarcinoma cells^14^. For treatment response DTL and UBE2S may interact with key cell cycle regulators and DNA repair proteins, thereby affecting the cellular response to chemotherapy drugs. DTL degrades CDT1 (a DNA replication licensing factor), thereby preventing abnormal DNA replication under replication stress and maintaining genomic stability^57^. In platinum-based chemotherapy (such as cisplatin), the loss of DTL function may weaken the DNA damage response (DDR), leading to the accumulation of unrepaired DNA adducts, which ultimately enhances chemotherapy sensitivity through the apoptosis pathway^58^. UBE2S collaborates with UBE2C to regulate the Anaphase-Promoting Complex/Cyclosome (APC/C). By extending ubiquitin chains, it promotes the degradation of cell cycle proteins such as Cyclin B1, thereby maintaining the mitotic process. The absence of UBE2S can lead to abnormal chromosome segregation and genomic instability, which in turn enhances the sensitivity to microtubule inhibitors like paclitaxel^59^. Additionally, UBE2S might play a role in the tumor microenvironment’s immune response, thereby affecting the efficacy of immunotherapies^14^. CISH binds to and degrades PLC-γ1 through its SH2 domain, thereby inhibiting calcium signaling following TCR activation and consequently reducing the release of IFN-γ and TNF-α^60,61^. High CISH expression may thus create an immunosuppressive microenvironment, reducing the efficacy of immune checkpoint inhibitors (ICIs) by inhibiting cytotoxic T-cell activation.

The immune infiltration, TMB, immune checkpoint gene expression profile, medication response, and KEGG score were all compared between the groups with high and low URRS. According to the results, the high URRS group had higher level immunotherapy signatures, including upregulated PD1/L1 expression level^62^, higher TMB^63^, TNB^64^, and CD8 + T cell abundance^65^, immune-related KEGG pathways such as mismatch repair pathway^66^ and a higher proportion of CR/PR in IMvigor 210 cohort, while lower IC50 value in chemotherapy drugs^46^. These results indicated that the high URRS group might be benefited from immune-related drugs and chemotherapy (such as including cisplatin, vinblastine, paclitaxel, and docetaxel) and the low URRS group might have better response from target drugs (including Selumetinib and Axitinib). By examining the pathways and biological processes these genes are involved in, we discuss their potential roles in tumor development, metastasis, and resistance to therapy. This analysis emphasizes the relevance of ubiquitination in the molecular pathology of lung adenocarcinoma and its potential as a therapeutic target. In the high URRS group, we observed a higher abundance of immune cells such as CD8 + T cells and macrophages, which are known to play key roles in tumor immune surveillance. Additionally, the upregulation of immune checkpoints such as PD-1 and PD-L1 suggests that these patients may benefit from immunotherapy. Mechanistically, the activation of immune pathways like mismatch repair and homologous recombination repair further supports the association between the high URRS group and an enhanced immune response.

The lung adenocarcinoma risk score model in this study is superior to the prognostic models in some previous studies. Wu et al.^67^ screened five m6A-related genes to form a prognostic signature, but the maximum AUC of their training dataset was only 0.684, which was lower than the maximum AUC in our study, and was only validated in two independent external validation datasets, whereas the present study obtained promising results in all six validation datasets. A recent study^68^ successfully utilized various screening approaches to identify four immune-related genes that form a prognostic model for predicting the prognosis of patients with lung cancer. Nevertheless, the highest AUC achieved by the training dataset was 0.669, while the prognostic model only reached a maximum AUC of 0.605 (1-year) when validated with the GSE68465 validation dataset. However, when validated against the GSE68465 dataset using URRS, the maximum AUC increased to 0.681 (1-year). Compared to the study^69^, which also constructed a risk model for breast cancer, our study employs a more comprehensive gene selection process by integrating LASSO Cox regression and Random Survival Forest algorithms. This results in improved model robustness and validation across multiple independent datasets, providing stronger prognostic accuracy. The relationship between ubiquitination genes and survival in patients with lung adenocarcinoma has also been investigated in previous studies. A five-gene signature involving ubiquitination has been developed by Xu and colleagues for estimating overall survival in patients having lung adenocarcinoma, while another recent study also developed a gene signature that relates to ubiquitination which predicts patient prognosis, immunological characteristics, and therapeutic responses^54,70^. However, in these two studies, the authors simply intersected the screened genes with a limited range of ubiquitin-related genes, or just screened for genes from ubiquitin-related genes. Compared with previous studies, we have more comprehensively exploited the expression information of ubiquitin-associated genes by clustering and tagging patient subtypes with different ubiquitination characteristics, and then searching for potential differential markers from them.

The URRS model we developed has the potential to be integrated into current clinical practice for lung adenocarcinoma (LUAD) patients. Specifically, the URRS model can serve as a prognostic tool to identify high-risk and low-risk patients, thereby guiding personalized treatment strategies. For example, patients with high-risk scores could be monitored more closely or considered for aggressive treatment regimens, while those with low-risk scores might benefit from less intensive treatments. High URRS patients, who are associated with worse prognosis, higher tumor mutation burden (TMB), and elevated immune-related markers, may benefit more from immunotherapy and chemotherapy. In contrast, low URRS patients might be more suitable for targeted therapies. The four genes (DTL, STC1, CISH, and UBE2S) that constitute the URRS model can be explored as potential biomarkers for developing diagnostic or prognostic tests. These genes’ expression levels can be measured using RT-qPCR or other molecular detection methods. To translate these findings into clinically applicable tools, the next steps include experimental validation in independent clinical samples to confirm the correlation between URRS model gene expression levels and prognosis and treatment response. Additionally, multicenter studies should be conducted to validate the model’s applicability across diverse patient populations. If the URRS model demonstrates robust performance in clinical trials, it could be considered for incorporation into LUAD clinical guidelines to provide more precise treatment recommendations for clinicians.

However, our study does have certain drawbacks. Firstly, because clinical information and related genomic molecular information were derived from multiple publicly reported datasets, tumor heterogeneity between tumors or between patients is also unavoidable. Secondly, experiments are needed to validate the association between URRS and other genomic signatures in lung adenocarcinomas. Thirdly, the selection of cutoff values for URRS should be discussed in greater depth. The median was used as the cutoff in this study, and this method is not necessarily the most appropriate. Fourthly, while our analysis revealed significant associations between URRS and immune-related markers such as PD1/L1 expression, we acknowledge that we do not have direct data on immunotherapy response. This limitation highlights the need for further research to explore the relationship between URRS and immunotherapy outcomes. The reliance on publicly available datasets such as TCGA and GEO may introduce selection bias. These datasets do not fully represent all lung adenocarcinoma patient populations. Moreover, while bioinformatics methods provide a solid foundation for gene identification, experimental validation of these ubiquitination-related genes (URGs) is necessary to confirm their biological relevance. Future research should focus on validating these findings in clinical samples and understanding the underlying mechanisms.

The applicability of the URRS model may be influenced by tumor heterogeneity, particularly differences in subtypes and stages. Our study primarily focused on LUAD patients, constructing and validating the URRS model within LUAD cohorts. However, we observed that the expression of the four genes in the URRS varied with tumor stages (Fig S2a-d), indicating that the prognostic ability of the URRS may differ across stages. Additionally, while the URRS model demonstrated consistent roles in pan-cancer prognosis (Fig. 6c), the specific impact of LUAD subtypes, such as acinar, papillary, micropapillary, and solid variants, was not explicitly addressed. These subtypes have distinct genetic and phenotypic profiles, which could affect the model’s predictive accuracy. Therefore, the URRS model’s applicability may be limited in certain subtypes or stages, highlighting the need for further stratified analyses to better understand its performance across diverse patient groups.

## Conclusion

In conclusion, our research established a URRS signature consisting of 4 ubiquitination-related genes, DTL, STC1, CISH, and UBE2S. This is a valid prognostic profile for LUAD and correlates with the efficacy of chemotherapy and immunotherapy in patients. In addition, patients with LUAD who were classified into different groups by this prognostic profile benefited from different therapeutic agents., the URRS signature may help contribute to a more personalized treatment, which might benefit the outcome of LUAD patients.

## Electronic supplementary material

Below is the link to the electronic supplementary material.

Supplementary Material 1

Supplementary Material 2

Supplementary Material 3

Supplementary Material 4

Supplementary Material 5

Supplementary Material 6

Supplementary Material 7

## Data Availability

The data used to support the findings of this study are available at cBioPortal database (https://www.cbioportal.org/study/summary?id=luad_tcga_pan_can_atlas_2018), UCSC Xena (https://xenabrowser.net), and NCBI GEO database (https://www.ncbi.nlm.nih.gov/geo/, accession numbers: GSE30219, GSE37745, GSE41271, GSE42127, GSE68465, and GSE72094.
