# Supplementary material for: Construction of ubiquitination-related risk model for predicting prognosis in lung adenocarcinoma
Source: Sci Rep. 2025 Apr 6;15:11787. doi: 10.1038/s41598-025-92177-4 (PMC11973225; doi:10.1038/s41598-025-92177-4)
Supplement: Supplementary file 1 — Supplementary Material 1 [file 41598_2025_92177_MOESM1_ESM.docx]

# Supplementary Material

**Supplementary Figure S1.** The ROC curves in training and validation cohorts. **(a)** TCGA cohort. **(b)** GSE32019 cohort. **(c)** GSE37745 cohort. **(d)** GSE41271 cohort. **(e)** GSE42127 cohort. **(f)** GSE68845 cohort. **(g)** GSE72094 cohort.

**Supplementary Figure S2.** Expression level of four genes in different stages. **(a)** CISH. **(b)** DTL. **(c)** STC1. **(d)** UBE2S. **(e)** Correlation between methylation and mRNA expression of four genes in LUAD.

**Supplementary Figure S3.** TIDE score and ESTIMATE score in LUAD. **(a)** Dysfunction score. **(b)** Exclusion score. **(c)** Immune score. **(d)** Stromal score. **(e)** ESTIMATE score.

**Supplementary Table 1.** Datasets information.

**Supplementary Table 2.** The proportion of censored data in all datasets.

**Supplementary Table 3.** The RNA expression data of four URGs using RT-qPCR experiment.
