# Supplementary figures and images for: Construction of ubiquitination-related risk model for predicting prognosis in lung adenocarcinoma
Source: Sci Rep. 2025 Apr 6;15:11787. doi: 10.1038/s41598-025-92177-4 (PMC11973225; doi:10.1038/s41598-025-92177-4)

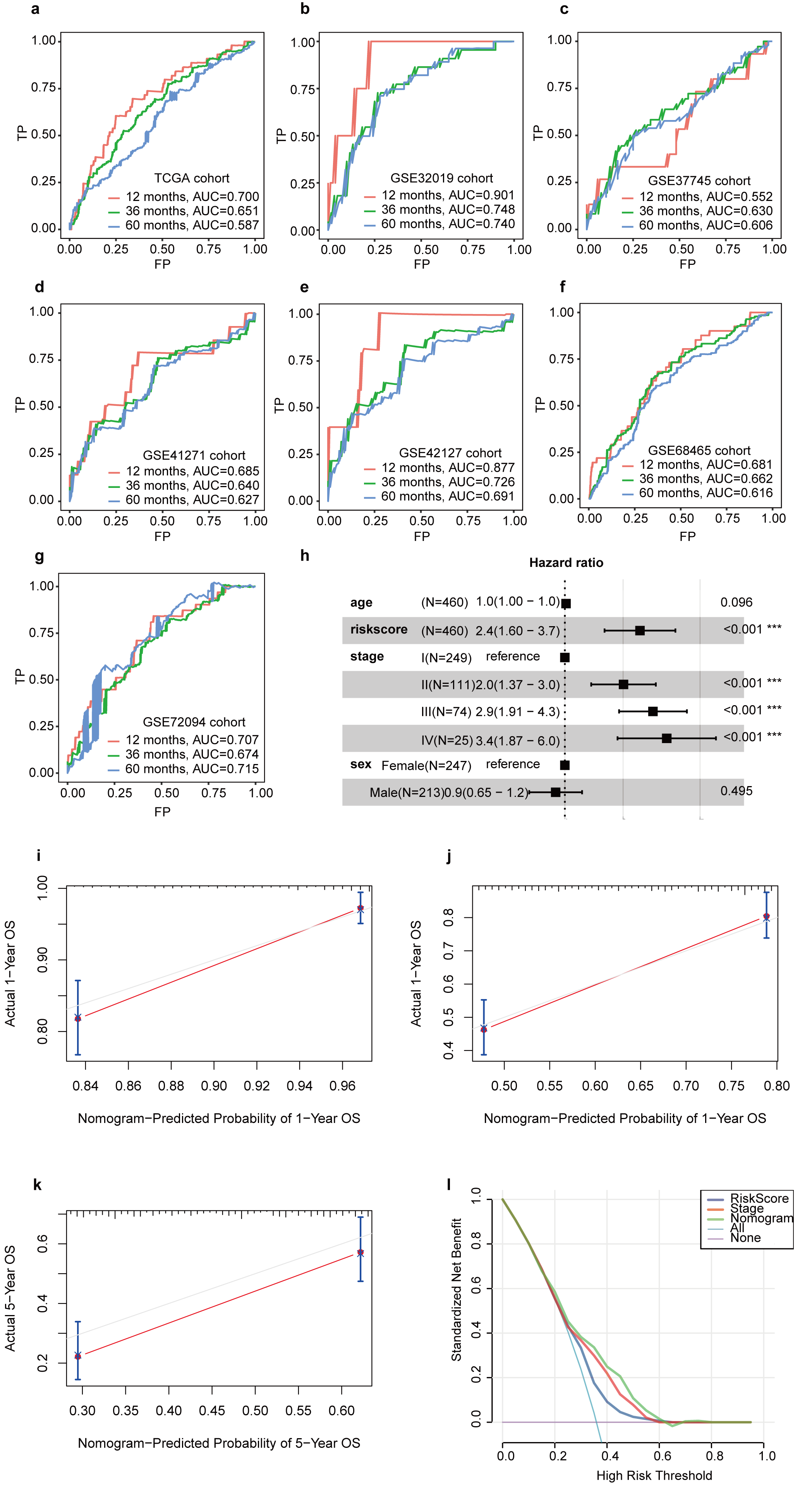

Supplement: Supplementary file 5 — Supplementary Material 5 [file 41598_2025_92177_MOESM5_ESM.tif]

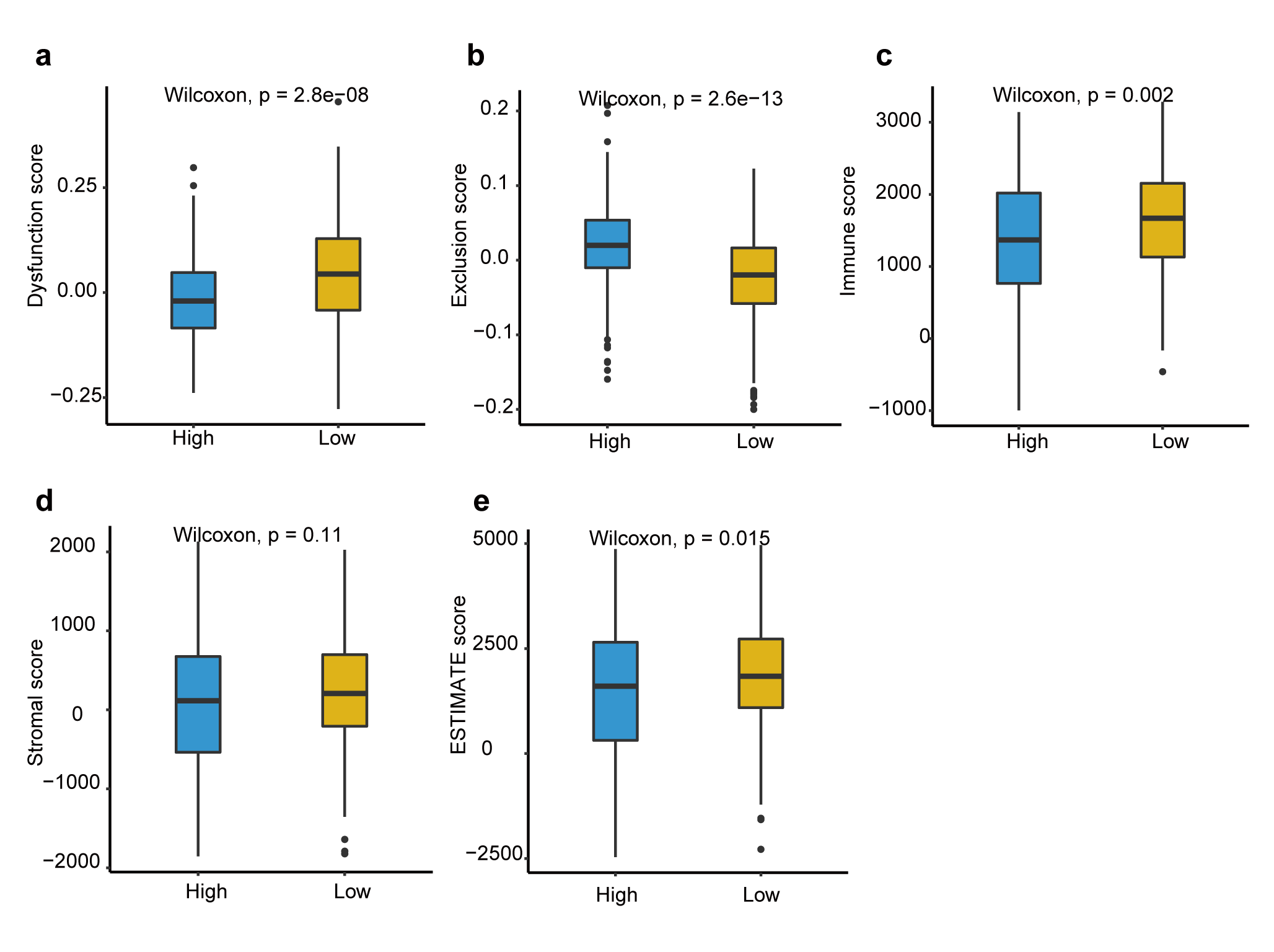

Supplement: Supplementary file 6 — Supplementary Material 6 [file 41598_2025_92177_MOESM6_ESM.tif]

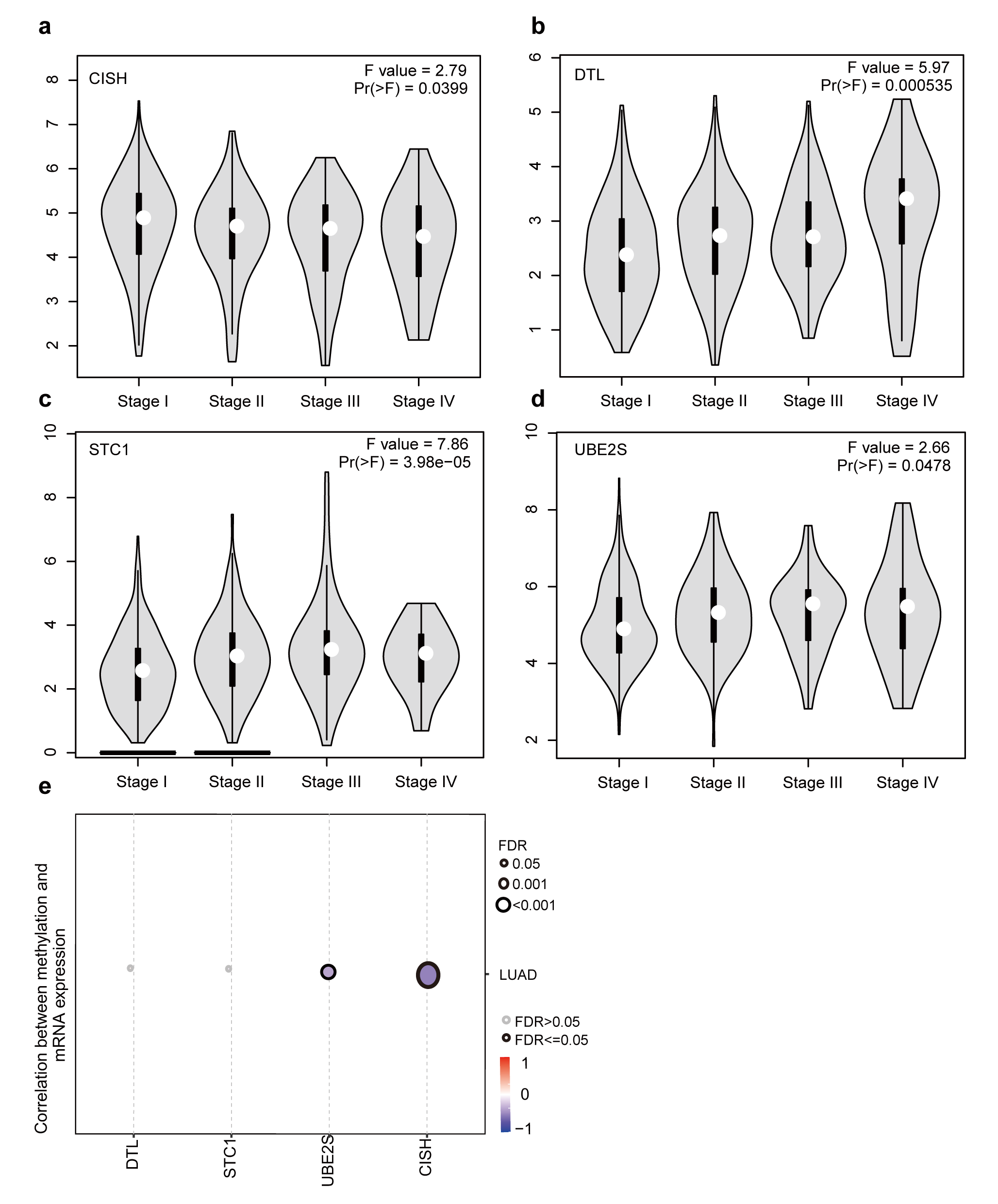

Supplement: Supplementary file 7 — Supplementary Material 7 [file 41598_2025_92177_MOESM7_ESM.tif]
